# A Laboratory Based Investigation of a New Elastic Toothbrush Head

**DOI:** 10.1155/2014/763968

**Published:** 2014-11-18

**Authors:** Lorenzo Mazza, Maria Rosaria Gatto, Giuseppe Monaco, Gian Andrea Pelliccioni

**Affiliations:** Department of Biomedical and Neuromotor Sciences, Alma Mater Studiorum-University of Bologna School of Dentistry, Via San Vitale 59, 40125 Bologna, Italy

## Abstract

*Purpose*. To demonstrate the noninferiority of a new toothbrush head with retractile bristles compared to traditional toothbrush heads on dental models. *Methods*. The new toothbrush head, mounted on manual and electrical handles, presents retractile bristle groups that can singularly retract over its entire length and offer calibrated resistance. Fourteen gypsum models of dental arches, twelve with and two without anatomical impairments, were spread with a “plaque simulator.” Each arch was brushed twice with each of the four toothbrushes, one minute by the same operator, blinded to the study. The plaque index (PI) was recorded at the end of each brushing session. GLM for repeated measures analysed the data. *Results*. On all the casts, the manual prototype and the electric prototype, removed 11% and 14% more “plaque simulator” compared to the standard toothbrush. In presence of dental anomalies, the prototypes removed 13% and 16% more plaque, respectively, compared to standard toothbrushes (*P* = 0.04). In both situations, the 95% confidence intervals of PI did not include −10% (the minimal margin of clinical relevance). *Conclusions*. The prototype is more effective in removing plaque from the casts with anomalies. The noninferiority of the prototype with respect to the standard toothbrushes was demonstrated.

## 1. Introduction

The prevention of periodontal diseases and carious lesions is based on maintaining good oral hygiene [1].

Many studies focused their efforts on different types of toothbrushes, but despite the improvements in developing innovative toothbrushes, they remove, on average, only approximately 50% of plaque [2, 3]. During brushing, optimal cleaning results when the bristles work with their ends directly on the tooth enamel with limited pressure. However, in the presence of dental braces, tooth crowding, rotated teeth, diastema, missing teeth, and all of the situations in which strong differences in the teeth profile is present, this technique is inefficient [4]. In reality, the bristles either bend, working on the teeth with their lateral surface, or do not touch the tooth surface at all, reducing the brushing effectiveness and avoiding optimal cleanliness of the teeth [5].

Different shapes of the handle and head of the toothbrush were proposed to increase plaque removing efficiency in hard-to-reach places [2, 6].

The optimal brushing pressure on the teeth should not exceed 300–400 g [7]. However, most people apply greater force, thinking that greater pressure will result in increased dental plaque removal. The excessive pressure does not improve oral hygiene [8] but, with time, causes brushing lesions in the form of gingival recessions and abrasions at the dental neck [9], even when correct brushing is used [10]. In subjects with a thin gingival biotype, or after an oral surgery, brushing lesions contrast the necessity of respecting the periodontium. Aiming to reduce the risk of lesions or recessions, many authors proposed modifications to the bristles or to the mechanism of their adaptation to the tooth surface. A recent study by Heasman et al. [10] faced this problem by testing a controlled pressure system incorporated in a powered toothbrush.

The purpose of this study is to compare the effectiveness of the plaque removal simulator on dental models using a new type of toothbrush head with retractile bristles versus a traditional toothbrush head in vitro. The research hypothesis was that the difference in the reduction of the plaque index between the prototype and the standard toothbrush, either manual or electric, was ≤10% (clinical relevance margin). The null hypothesis was that this difference was >10%.

## 2. Materials and Methods

### 2.1. Materials

**Figure 1:**
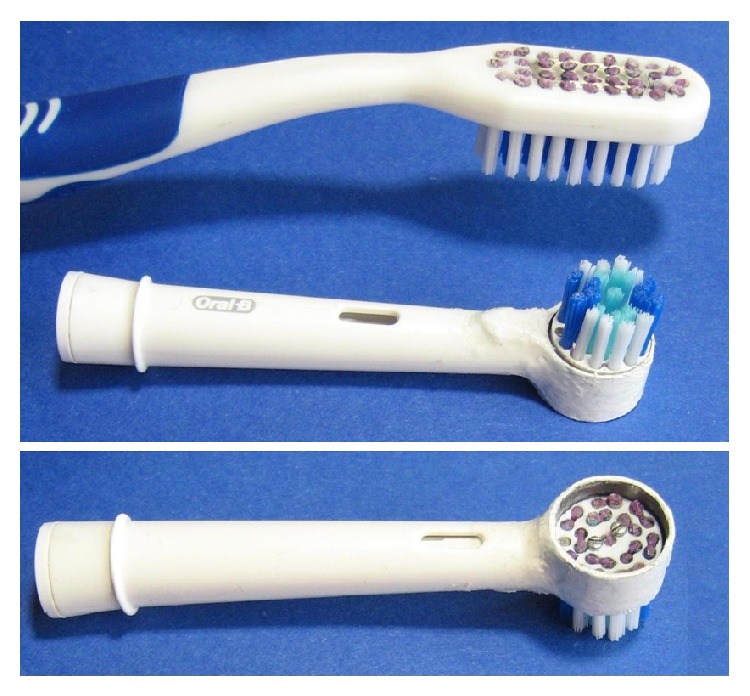
Manual toothbrush and electrical toothbrush head, modified with the proposed mechanism.

The innovation of the proposed toothbrush head consists of retractile bristles groups that can be inserted on both manual and electrical handles, and any number of bristles and any type of head geometry can be adapted. Each bristle group can singularly retract for its entire length, always offering calibrated resistance. The bristles can move in their holes and work under elastic action; they can slip inside the holes when pressed and then return to their original position when the pressure ends. The prototypes presented result from a modification of the manual toothbrush Silver Care Plus (Piave Spa, Onara di Tombolo, PD, Italy) and the head Oral-B Precision Clean (Procter & Gamble, Cincinnati, OH). The original head geometry and the number of bristles have been maintained in both cases (Figure 1).

The effectiveness in plaque removal for the two prototypes, manual and electric, was compared with that of the two original toothbrushes from which they have been derived.

### 2.2. Methods

Fourteen gypsum models of dental arches, twelve with different anatomical impairments (Table 1) and two without any abnormalities, were used.

A liquid “plaque simulator” made of gypsum and powder mixed with water [12] was applied with a small brush on all of the dental surfaces and on the marginal gum of each model.

Once dried, a thin layer of solid and compact material, similar to plaque, covered the models.

Each model was brushed with the manual prototype (modified Silver Care Plus). After the removal, the plaque simulator was reapplied, and the model was brushed with the standard manual toothbrush (Silver Care Plus) with a medium bristle type. The same procedure was performed for the electric toothbrush (Oral-B Triumph Professional Care 9000) with the modified experimental head (Modified Oral-B Precision Clean) and a standard electric toothbrush (Oral-B Triumph Professional Care 9000; Precision Clean head).

The tooth brushing technique for the manual devices was circular for all of the casts with dental braces, first on the cervical third and then on the coronal third of the tooth, as this condition requires, and the roller technique was used for the remaining casts. The tooth brushing technique recommended by the producer was used for the electric toothbrush. A single trained operator, blinded to the aims of the study, brushed each arch for one minute. The length of each session was 2 minutes because each procedure was repeated twice.

The main outcome was the plaque index (simplified O'Leary) [13] recorded after each treatment session.

The calibration of the plaque simulator applied on each model was performed by putting it into a porcelain bowl that was weighed before each application and was completely emptied afterwards. Intraobserver variation was evaluated by measuring the plaque index three times, at intervals of 5 minutes, by using either the standard or prototype toothbrush on 5 models with anomalies and 5 without anomalies (ICC = 0.985, *P* = 0.0001).

The experimenter who undertook brushing was calibrated by brushing the models ten times (5 on casts with anomalies and 5 on casts without anomalies) using either the standard or the prototype.

### 2.3. Statistical Analysis

A noninferiority trial was planned by hypothesizing a margin of clinical relevance in the plaque index (PI) reduction of 10%, which was set on the basis that a value of 20% of the plaque index is the clinically relevant level that indicates good oral hygiene. With a power of 80% and a standard deviation of 1%, a sample size of 2 observations for each dental cast was planned at an *α* level of 0.05. Based on the Kolmogorov-Smirnov test, the distribution of PI did not significantly differ from the Gaussian. Consequently, PI data were presented as a mean (SD). The comparisons between the prototype and the standard toothbrush were performed using a GLM for repeated measures. One-sided 95% confidence intervals were computed, with the aim to evaluate the noninferiority of the prototypes (both manual and electric) in comparison with the standard toothbrushes. The *α* level was set at 0.05.

## 3. Results

With all of the dental arch models, on average, the manual prototype removed 11% more “plaque simulator” compared to the standard toothbrush, while the prototype head mounted on the electrical toothbrush removed 14% more plaque compared to the standard electric toothbrush (Table 2).

No significant differences in the PI were obtained by comparing the two types of toothbrushes; however, the 95% confidence intervals of the PI did not include −10% (the minimal margin of clinical relevance).

When only considering the 12 models with anatomical problems, the prototypes removed, on average, 13% and 16% more plaque, respectively, compared to the standard manual and electric toothbrushes.

Significant differences in the PI were obtained by comparing either manual or electric devices, proving a greater efficiency of the prototypes (*P* = 0.04). Moreover, in this case, the 95% confidence intervals of PI did not include the minimal margin of clinical relevance, both for manual and electric devices (Table 3).

Significant differences were observed between repeated measures (*P* = 0.03).

**Figure 2:**
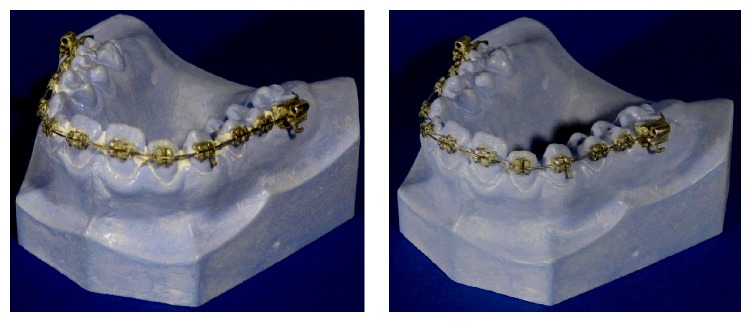
“Plaque simulator” residuals on the orthodontic model number 5, after manual brushing. On the left side the results for the normal toothbrush, on the right side the prototype results.

The best results were obtained in presence of dental braces (Figure 2). The prototype head improved the removal efficiency to 25% for the manual and 34% for the electric devices.

## 4. Discussion

The aim of this noninferiority study was to compare toothbrush prototypes, both manual and electric, with standard toothbrushes and verify the ability of the prototypes to support the patients with specific anatomical situations when oral hygiene is particularly critical.

Our results, with the limit of an in vitro study, demonstrated that the experimental devices are not inferior to the standard ones. Moreover, the prototype head mounted on the electric toothbrush was significantly more effective compared to the standard electric toothbrush when anatomical anomalies were present. Significant differences observed between repeated measures (*P* = 0.03) could be explained by the difficulty in reproducing similar PI values on casts with anatomical anomalies.

A lacune in this in vitro experimentation is the lack of control on the pressure exercised by the prototypes and the standard toothbrushes because no sensors were positioned on the models. A recent systematic review focused on specific variables associated with tooth brushing such as pressure, time spent brushing, bristle type (stiffness and end-shape), filament characteristics, or the use of a dentifrice [13].

However, our interest in this phase was exclusively to determine the ability of the devices in removing the plaque simulator. Further extensions of the study will measure pressure under real application conditions. In the last four years, many studies were conducted on experimental toothbrushes to evaluate their efficacy in removing plaque by comparing powered toothbrushes to manual toothbrushes [14–18]. A recent systematic review [5] suggested the superiority of certain modes of powered over manual tooth brushing for plaque and gingivitis reduction, although direct comparison between different modes of powered tooth brushing was not allowed, indicating that further trials for good quality need to establish the superiority of certain modes over other modes.

In the present study, our efforts were aimed at modifying the adaptation of the bristles of the toothbrush to different anatomical situations [19], rather than modifying its power mechanism.

Other studies evaluated the influence of the characteristics of the bristles on the efficacy of the toothbrush in plaque removal, finding that medium toothbrushes have a greater ability to remove biofilm than soft toothbrushes [20]. Toothbrushes with elongated fine bristles and a vibrating bristle field were consistently and statistically superior to the conventional ones [21] in accessing interproximal sites and removing simulated plaque. Single-tufted toothbrushes on the posterior molars were more effective than flat-trimmed bristles [22]. Toothbrushes with extended bristles were more effective than toothbrushes with x-angled or flat multitufted bristles in removing artificial plaque from interproximal sites [23]. Toothbrushes with an advanced crisscross bristle design were superior to toothbrushes with straight bristles [5]. A multilevel manual toothbrush compared with a control flat-trimmed manual reference toothbrush was superior, although the difference is clinically considered to be small, and the amount of remaining plaque was not significantly different between brushes [24]. A uniquely shaped tapered-bristle manual toothbrush was more effective in the interproximal areas, at the gingival margin, and subgingivally compared to a toothbrush with bristles uniform in height and diameter [25]. Finally, toothbrushes with a tapered and cross angled soft bristle design were found to be more effective in removing dental plaque and reducing gingival inflammation than the ADA standard toothbrush [26]. Bypassing the different opinions on the most efficient type of bristle, the elastic head of the prototypes used in our study can support every type of bristle.

In conclusion, the noninferiority of the prototype to the standard toothbrush in removing plaque suggests its applicability on the mouth of patients, and its superiority in the presence of anomalies suggests that every single bristle group could reach the dental surface, even under difficult oral-hygiene conditions. However, clinical randomized trials, in the situation of controlled pressure, are needed to confirm in vivo the experimental results.

## Figures and Tables

**Table 1:** Description of each tested model.

Gypsum model	Description
1	Upper bridge 13–16 and 23–25; **diastema 12-11-21-22.**
2	Lower bridge **34–37 and 44–47.**
3	Severe chronic periodontitis: **clinical crown lengthening with the opening of dental spaces and many diastemata; 27 furcation exposed; 23 inclined; 26 missed.**
4	Severe chronic periodontitis: **clinical crown lengthening with the opening of dental spaces and many diastemata; 46 furcation exposed; 36-37 missed.**
5	Orthodontic braces; **diastemata 12-13-14 and 23-24.**
6	**13-12-21-22 rotated for** crowding of the teeth; **16 slightly extruded.**
7	Crowding of the teeth 34,…, 44**, 33 highly** displaced; **43 missed; 36 slightly extruded with** enlargement of interdental space; **furcation exposed on 36 and 46.**
8	**Wide diastemata between all teeth.**
9	**Diastemata 35-36, 33-34, 42-43, and 43-44.**
10	Crowding of the teeth 33,…, 43**; 32 highly** displaced.
11	Orthodontic braces.
12	**Crown on 36; bridge 44–47; 34 missed;** periodontitis **with** gingival recessions; **32-33 lingual inclined. **
13-14	**Ideal dental disposition. Absence of any anatomical variations; all teeth present except the third molars.**

**Table 2:** % PI for type of toothbrush for the 14 casts (% PI data).

Type of brushing	Manual		Electric	
Type of toothbrush	Standard	Prototype	Standard	Prototype
	Mean (standard deviation)	Mean (standard deviation)	Mean (standard deviation)	Mean (standard deviation)

1st observation	37.21 (20.56)	25.29 (14.93)	34.14 (18.38)	19.93 (11.59)
2nd observation	37.50 (18.64)	28.00 (15.88)	33.79 (17.29)	20.50 (10.78)
Total	**37.36 (19.26)**	**26.6 (15.19)**	**33.96 (17.51)**	**20.21 (10.98)**

GLM for repeated measures	Measures *F* = 3.96 *P* = 0.06			
	Toothbrush *F* = 2.61 *P* = 0.12			

Mean difference of PI between devices	11%		14%	
95% CI of the difference	−3%–24%		2%–25%	

**Table 3:** Comparison of PI between prototype and toothbrush, on the 12 casts with anomalies (% PI data).

Type of brushing	Manual		Electric	
Type of toothbrush	Standard	Prototype	Standard	Prototype
	Mean (standard deviation)	Mean (standard deviation)	Mean (standard deviation)	Mean (standard deviation)

1st % PI observation	42.42 (17.07)	28.08 (14.23)	38.83 (15.21)	22.67 (10.07)
2nd % PI observation	42.92 (13.63)	31.42 (14.38)	38.83 (12.61)	22.92 (9.62)
Total	**42.67 (15.11)**	**29.75 (14.09)**	**38.83 (13.66)**	**22.79 (9.63)**

GLM for repeated measures	Measures *F* = 5.191 *P* = 0.03			
	Toothbrush *F* = 4.61 *P* = 0.04			

Mean difference of PI between devices	13%		16%	
95% CI of the difference	0.4%–26%		6%–26%	
